# Application of a Digital Mental Health Clinic in Secondary Schools: Functionality and Effectiveness Evaluation

**DOI:** 10.2196/46494

**Published:** 2023-10-26

**Authors:** Yi Xu, Hongshen Yang, Zhou Jin, Jian Xiang, Haiyun Xu, Yili Huang Pokay, Haibo Mao, Xugong Cai, Yili Wu, Deborah Baofeng Wang

**Affiliations:** 1 Zhejiang Jerinte Health Technology Co, Ltd. Wenzhou China; 2 Zhejiang Provincial Clinical Research Center for Mental Disorders The Affiliated Kangning Hospital Wenzhou Medical University Wenzhou China; 3 Key Laboratory of Alzheimer’s Disease of Zhejiang Province School of Mental Health and The Affiliated Kangning Hospital Wenzhou Medical University Wenzhou China; 4 Lyons Insights Consulting, LLC Lyons, IL United States

**Keywords:** adolescents, digital mental health clinic, emotional distress, mental health service, secondary school

## Abstract

**Background:**

Adolescents experience relatively more stress than other populations as they are facing rapid physical changes and adapting to complex social environments. However, access for this population to professional service providers is limited. Therefore, there is an increasing need for access to mental health services and new mental health care resources tailored to adolescents.

**Objective:**

The aim of this study was to evaluate the functionality and effectiveness of a school digital mental health clinic (DMHC) created by a Chinese psychiatric hospital and provided to secondary school students for a trial.

**Methods:**

The trial period of the DMHC was from January to July 2021 at three secondary schools in Taizhou City, China. Under a collaborative agreement between the local educational bureau and provider, use of the DMHC was free to all students, teachers, and staff of the schools. The functionality of the DMHC was compared with existing digital health interventions introduced in the literature and its effectiveness was quantitatively analyzed in terms of the volume of received counseling calls, number of calls per 100 students, length and time of calls, and reasons for the calls. The mini course video views were analyzed by topics and viewing time.

**Results:**

The design functions of the DMHC are well aligned with required factors defined in the literature. The first advantage of this DMHC is its high accessibility to students in the three schools. All functions of the DMHC are free to use by students, thereby eliminating the economic barriers to seeking and receiving care. Students can receive virtual counseling during or after regular working hours. Acceptability of the DHMC was further ensured by the full support from a national top-tier mental health facility. Any audio or video call from a student user would connect them to a live, qualified professional (ie, a psychiatrist or psychologist). Options are provided to view and listen to resources for stress relief or tips to help address mental health needs. The major reasons for the counseling calls included difficulties in learning, interpersonal relationships, and emotional distress. The three topics with the highest level of interest for the mini course videos were emotional assistance, personal growth, and family member relationships. The DMHC served as an effective tool for crisis prevention and intervention during nonworking hours as most of the live calls and mini video viewing occurred after school or over the weekend. Furthermore, the DMHC helped three students at high risk for suicide and self-injury through live-call intervention.

**Conclusions:**

The DMHC is an effective complementary solution to improve access to professional mental health care facilities, especially during nonworking hours, thereby helping adolescents meet their mental health needs. Extension of the DMHC into more schools and other settings is recommended.

## Introduction

### Background

Mental health problems among children and adolescents are of great significance because of their high prevalence, early onset, and heavy impact on the rest of a young person’s life. The percentage of children and adolescents in the United States who experience a mental disorder in a given year ranges from 13% to 20% [1]. The worldwide pooled prevalence of mental disorders was estimated at 13.4% (95% CI 11.3-15.9), with a prevalence of 6.5% for any anxiety disorder, 2.6% for any depressive disorder, 3.4% for attention-deficit/hyperactivity disorder, and 5.7% for any disruptive disorder [2]. In China, the average prevalence of any child mental disorder is 15.2% and that of a behavioral disorder is between 8.3% and 27.2% [3]. The latest estimated prevalence of nonsuicidal self-harm in middle-school students (aged 13-18 years) in China is 27.4%, which is much higher than the worldwide estimate of 19.5% [4]. During the COVID-19 pandemic, the prevalence rates of insomnia, depressive, and anxiety symptoms were 37.8%, 48.2%, and 36.7%, respectively, among Chinese adolescents [4]. In spite of the high occurrence of mental health problems, less than half of the youth with mental disorders receive medical treatment [5]. More specifically, 1 in 5 teenagers experiences depression, and two-thirds of teenagers with depression remain undiagnosed [6]. Poor mental health in early years tends to induce health problems and life issues in adulthood [7].

The large gap between the high prevalence and low diagnosis rates of mental health conditions is closely related to the scarcity of mental health resources in China [8]. There are fewer than 500 full-time child psychiatrists in the entire country and the geographic distribution of child psychiatrists is highly uneven, with no specialist child psychiatry services in many areas other than large cities such as Shanghai or Beijing [8]. According to Shi [9], only 34.8% of schools had school psychologists who were certified as mental health counselors or who had degrees in psychology and 19.6% of schools did not have any, even those working part time, with systematic psychological training. The scarcity of mental illness treatment resources, along with other barriers generally present globally, calls for alternative methods. One solution is digital health interventions (DHIs), which are health services delivered electronically via digital technologies such as smartphones, websites, SMS text messaging, virtual reality, digital games, and apps. 

### Adolescents Are Interested in DHIs

Research findings have identified the major barriers to help-seeking for youth with mental conditions. These include concerns about stigma and confidentiality, shame and embarrassment [10], poor mental health literacy, low awareness, preference for self-reliance [11], financial costs, and limited access [11]. In general, DHIs have proven to be effective means of providing mental health care [12]. The World Health Organization conducted a survey of 15,000 mobile health apps and discovered that 29% of them focus on mental health diagnosis, treatment, or support [13]. The UK National Health Service and the US National Institute of Mental Health have identified mental health apps as cost-effective and scalable solutions to addressing the mental health treatment gap. Prior literature proposed that mental health apps indeed have value in providing psychological treatment [14]. The promise of DHIs as effective tools is based on their potential to eliminate some of the barriers identified above because of the advantages identified by Khanna and Carper [15]: cost savings, availability, accessibility, treatment integrity, personalized treatment, rehearsal, and data gathering.

With respect to children and adolescents, DHIs are of particular interest for mental health treatment [16] and help-seeking [17]. In a study conducted among male adolescents, Clark et al [17] identified that the primary facilitators for help-seeking were increasing the accessibility of school-based mental health literacy programs and providing a wider range of formal and informal help-seeking options. Other identified facilitators were related to amendments in how mental health information is presented and investments into high-speed/low-effort help-seeking options. The first factor is related to young people’s high level of digital literacy [18,19]. DHI apps featuring functions appropriate for the digital literacy skills of children and adolescents have the potential to engage this population in mental health prevention and intervention activities [16]. Moreover, the results of a meta-analysis by Podina et al [20] demonstrated that minimal therapist involvement, rather than significant therapist involvement, was more beneficial for youth. Their findings suggest that DHIs, which offer flexibility in the level of therapist involvement, could be an acceptable intervention method for young people.

Given the advantages of DHIs to remove many of the existing mental health treatment and help-seeking barriers, and the fact that adolescents are most likely to receive mental health services in schools compared to other settings [21], a DHI tool was created and provided to secondary schools in Taizhou City, China, with the functions of a digital mental health clinic (DMHC), including (1) audio consultation, (2) video consultation, (3) psychological video mini courses, and (4) a link to an electronic hospital (e-Hospital) for mental health services. Unlike existing DHIs, this DMHC is installed physically in on-campus booths with touchscreens on the wall that allow adolescent students to access the services at any time in a private setting.

The aim of this study was to perform a comprehensive evaluation of the functionality and effectiveness of this DMHC. We also identified its unique strengths after comparing its key features with similar tools available in China and internationally. These features, including real-time video or audio calls to mental health professionals and round-the-clock digital resources for mental health literacy promotion and stress relief, are expected to help address the aforementioned concerns such as stigma and confidentiality and limited access to mental health services, thereby helping to solve the real-life mental health issues of secondary school students, serve their immediate needs, and increase their mental health literacy and help-seeking intentions.

## Methods

### Design Considerations of the DHMC

With reference to an established framework for a DHI defined by Liverpool et al [16], we took into account the following two themes and six factors when designing the DMHC. The first theme is “intervention-specific influences,” which determine the success of a DHI and involve the influencing factors of acceptability, usability, and suitability. Acceptability refers to users’ willingness to use the product; it is the functions and features of any tool that will largely encourage young people to use the intervention. These features entail certain images, specific language and appealing interfaces, videos, having less text, ability to personalize and to connect with others or receive text messages. Other features focus on whether the DHI is self-paced, user-friendly, age-appropriate, simple, and straightforward. Usability relies on clear and sufficient instructions on usage. Suitability consists of the degree to which a DHI is in line with daily activities, convenience, not having to travel for accessing the interventions, and ability to use it at home. The second theme is “person-specific influences,” which predict users’ engagement in DHI use relying on the three factors of motivation, opportunity, and capability. First, perceived need and usefulness lead to high motivation to use a DHI. Second, a user’s feeling of a sense of connectedness increases their opportunity to adopt the tool (ie, they are more likely to use the intervention if conversations with others, especially professionals, are facilitated). Finally, environmental pressure and government policies empower users with capability to use.

### Functions and Implementation of the DHMC

**Figure 1:**
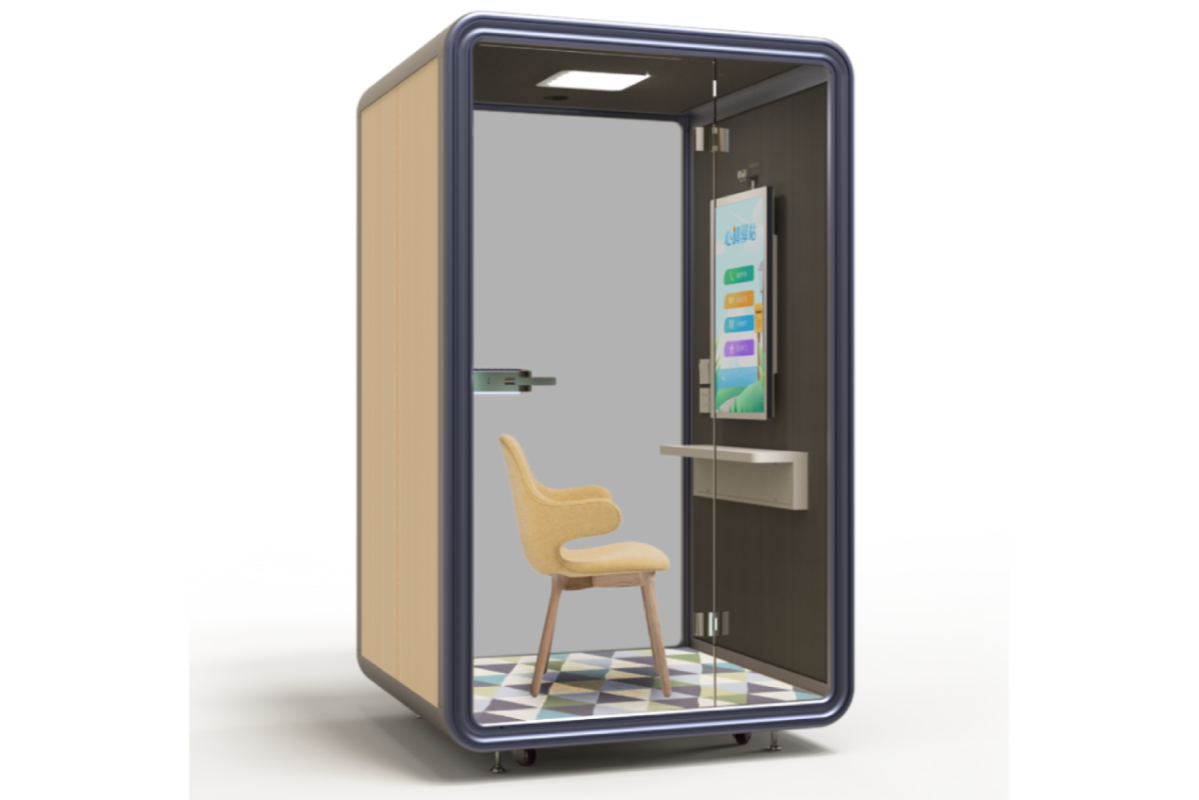
A 3D view of the digital mental health clinic (DMHC) established in a small booth. The DMHC functions through a touchscreen that is fixed onto the interior surface of one wall of a small booth. Four functional touch icons appear when the DMHC is in use; a user is free to choose among: (1) audio consultation, (2) video consultation, (3) psychological video mini courses, and (4) link to the electronic hospital for mental health services. Underneath the screen is a desktop attached to the same wall. Facing the wall, a chair is available for a client to be seated. The walls of the booth are made of soundproof materials.

The display screen of the DMHC is hung on one wall of a small booth with a chair available for one person. Below the display screen is a desktop attached to the same wall. The walls of the booth are made of soundproof materials. Figure 1 shows a 3D view of the DMHC. When the DMHC works, its display screen shows four functional touch icons for a user to choose from: (1) audio consultation, (2) video consultation, (3) psychological video mini courses, and (4) link to the e-Hospital for mental health services. The first two icons, when touched, connect a caller to a psychologist responsible for providing psychological consultation through an audio or video channel. The third icon takes a user to a list of video mini courses providing psychological education information, stress relief music, inspirational talks, and tips for better sleep. The fourth icon provides online service options for long-term mental health care offered by the psychiatric hospital. Under a collaborative agreement between the local educational bureau and the psychiatric hospital, three high schools in the city were selected, each with one DMHC installed. The trial period of the DMHC was between January and July 2021. All students in the schools had free access to the DMHC.

### Ethical Considerations

The Research Ethics Committee of The Affiliated Kangning Hospital of Wenzhou Medical University approved this study (YJ-2022-007-02).

The service starts with a statement shown on the display screen that indicates that the use of services provided by the DMHC will be analyzed for research and quality improvement purposes and that proceeding with the service constitutes consent. The materials used for the booth walls are sound-absorbing to protect the privacy of users and ensure a quiet place for a user to talk. All counseling calls are anonymous. Psychologists answering the calls have no information on the caller’s personal identification. The phone number of the caller is not displayed on the display screen and no call-back option is provided. In the event of a crisis (eg, suicide in action), the person who receives the call can reach out to an emergency contact staff member, who may take the necessary actions to prevent suicide or a disastrous event from occurring.

### Data Collection and Analysis

Data about the live calls and mini course videos recorded by the DMHC were collected for statistical analysis. The Shapiro-Wilk test was used to test the normality test of the data set. The *t* test or *χ*^2^ test with posthoc comparisons was performed to compare normally distributed data between the junior and senior high students using Excel and SPSS 26 software. Natural log transformation was performed for nonnormal data prior to the *t* test or *χ*^2^ test. The contents of all narrative records were summarized and analyzed by psychiatrists and psychologists who answered the calls.

## Results

### Functionality of the DMHC

The DMHC booth was located on the school campus, thus making its use convenient and increasing its accessibility, especially for those who live on campus. The touch icons on the display screen of the DMHC are straightforward, making the tool easy to understand and use, thus offering high usability. The greatest advantage of this tool compared to others is the full support from a national top-tier mental health facility. Touching any audio or video icon links a user to a qualified professional (ie, a psychiatrist or psychologist). Student users can receive a virtual counseling session during or after regular working hours. They also have the option to view and listen to resources for stress relief or tips to help address their mental health needs. The versatility of the tool ensures its acceptability.

### Live Calls by Students in the Three Schools

As indicated in Table 1, all three DMHCs received a total of 340 live calls during the trial period. Among these calls, 293 (86.2%) came from senior high students, whereas 47 (13.8%) came from junior high students. This substantial difference lies in the fact that the total number of students from senior high schools is significantly greater than that from the junior high schools. In fact, the proportions of the students using the DMHC in the two groups were comparable (5.2% vs 4.0%). Of all 340 calls, there were 285 (83.8%) audio calls, 50 (14.7%) video calls, and 5 (1.5%) invalid calls (ie, the caller did not proceed with the call after pressing the icon). It is of note that the junior and senior high school students showed differences in usage preference for audio and video calls. Compared to junior high students, a higher proportion of senior high students chose audio calls (85.7% vs 72.3%), but a lower proportion of senior high students preferred to use video calls relative to the junior high students (13.0% vs 25.5%). The proportion of invalid calls that occurred among junior and senior high students was comparable (2.1% vs 1.4%).

Overall, students received professional mental health services through the DMHC on campus for over 6000 minutes. Compared to junior high students, senior high students spent much more time using the DMHC (5792 minutes vs 427 minutes). This significant difference was not only due to the difference in the number of students between the two groups but also because the average duration per call among senior high students was longer than that of the junior high students (19.8 minutes vs 9.1 minutes). Furthermore, the record of time at calling showed that most (57.6%) of the calls occurred outside of working hours (ie, workday evening or weekend/holiday). Interestingly, the junior and senior high students showed significant differences in the time of calling. Specifically, most of the calls (61.3%) from senior high students occurred during nonworking hours, whereas most of the calls (67.4%) from junior students occurred during working hours.

**Table 1:** Live calls by junior and senior high school students (N=6855).

Category				Total		Juniors		Seniors		*P* value^a^	
Students, n (%)				6855 (100)		1175 (17.1)		5680 (82.9)		N/A^b^	
Calls, n (%)				340 (100)		47 (13.8)		293 (86.2)		N/A	
Calls/100 students				5.0		4.0		5.2		.11	
**Type of call, n (%)**											.01
	Audio			285 (83.8)		34 (72.3)		251 (85.7)			
	Video			50 (12.1)		12 (25.5)		38 (13.0)			
	Invalid			5 (2.6)		1 (2.1)		4 (1.4)			
Call duration, minutes (% of total)				6219 (100)		427 (6.9)		5792 (93.1)		N/A	
Average call duration (minutes)				18.3		9.1		19.8		<.001	
**Time calling, n (%)**											<.001^c^
	Working time			142 (42.4)		29 (67.4)		113 (38.7)			
	**Nonworking time**			193 (57.6)		14 (32.6)		179 (61.3)			
		Weekday evening	132 (39.4)		12 (27.9)		120 (41.1)		.10		
		Weekend/holiday	61 (18.2)		2 (4.7)		59 (20.2)		<.001		

^a^Comparison between junior and senior high school students using the *t* test or *χ*^2^ test.

^b^N/A: not applicable; statistical analysis not performed for these variables.

^c^Based on the comparison between calls made during working and nonworking hours.

### Reasons for Students to Call for Help

Students were driven by seven main reasons for making a call, as shown in Table 2. For all the callers, the order of afflicting problems was learning difficulties (30.5%), emotional distress (29.1%), interpersonal relationships (27.0%), personal growth (5.5%), family member relation (3.5%), choice of major (2.8%), and behavioral problems (1.7%). Learning difficulties were accompanied by test anxiety, attention issues, tiredness, low learning efficiency, lack of interest, and even physical symptoms (dizziness, nausea, and insomnia). Adolescents also expressed their struggles with low mood, depression, emotional loss of control, family death, broken relationship, test failure, irritability, emotional entanglement, loneliness, and peer pressure. An in-depth review of students’ verbatim comments revealed their desire, effort, and struggles in establishing and maintaining good relationships with classmates, teachers, parents, friends, siblings, and teammates. They also expressed their disappointment and loneliness when these relationships are not working as expected. This order was slightly different for junior and senior high students. Among senior high students, the order was learning difficulties (32.0%), emotional distress (28.9%), interpersonal relationship (25.0%), personal growth (6.3%), choice of major (3.1%), family member relation (2.7%), and behavioral problems (2.0%). Among junior high students, the top three afflictions were interpersonal relationships (42.4%), emotional distress (30.3%), and learning difficulties (18.2%). A relatively low proportion of this population suffered from family member relation problems (9.1%). They expressed no concerns in personal growth and behavioral problems. Note that students are not required to choose a major in junior high school.

**Table 2:** Reasons students called for help.

Reasons	Total (N=289), n (%)	Juniors (n=33), n (%)	Seniors (n=256), n (%)
Learning difficulties	88 (30.5)	6 (18.2)	82 (32)
Emotional distress	84 (29.1)	10 (30.3)	74 (28.9)
Interpersonal relationship	78 (27.0)	14 (42.4)	64 (25.0)
Personal growth	16 (5.5)	0 (0)	16 (6.3)
Family member relation	10 (3.5)	3 (9.1)	7 (2.7)
Choice of major	8 (2.8)	N/A^a^	8 (3.1)
Behavior problems	5 (1.7)	0 (0)	5 (2.0)

^a^N/A: not applicable; junior high school students are not required to choose a subject or major.

### Mini Course Video Views

Another pool of major resources provided by the current DMHC are a series of mini course videos. During the probation period, a total of 623 views were recorded, as shown in Table 3. The majority (88.0%) of the views were by senior high students as this group had many more members than the junior high group (5680 vs 1175) and a higher proportion of them watched mini course videos compared to junior high students (9.65 views/100 students vs 6.38 views/100 students). Out of all the video viewings, most of them (n=387, 62.1%) occurred at a nonworking time, while a small portion occurred at a working time (n=236, 37.9%). The junior and senior high students did not show a difference in the time of viewing.

We analyzed the contents covered by all the mini course videos watched by the students prior to comparing the junior high to senior high student groups. As shown in Table 4, videos containing information on emotional assistance (39.3%), personal growth (36.8%), and family member relationships (6.7%) were the most attractive to all students. For junior high students, these figures changed to 25.3%, 24.0%, and 24.0%, respectively, with no significant difference across the three categories. However, senior high students focused their concerns on emotional assistance (41.2%) and personal growth (38.5%), but not on family member relationships (4.4%). Interestingly, both the junior and senior high students did not pay substantial attention to issues relevant to youth mental health. However, a significant difference was found between the junior and senior high students in viewing videos covering interpersonal relationship issues (ie, junior high students showed more concern related to interpersonal relationships than the senior high students; 5.3% vs 1.1%).

Issues described by students that fall under the “others” category entailed a variety of topics, including homosexual legalization, physical examination challenges (fear of blood drawing and getting shots), concerns over emotional attachment to a desk mate, being attracted by the psychologist’s voice and wanting to meet them, concerns over improper behaviors, bad dreams, concerns over teachers’ attitudes, sleep problems, questions about their own psychological condition after reading psychology books, dissatisfaction about academic pressure and deprivation of weekend and break time, fear of being left behind academically, and worry about memory loss.

**Table 3:** Mini course video views by junior and senior high school students (N=6855).

Category			Total	Juniors		Seniors		*P* value	
Students, n (%)			6855 (100)	1175 (17.1)		5680 (82.9)		N/A^a^	
Video views, n (%)			623 (100)	75 (12.0)		548 (88.0)		N/A	
Views/100 students			9.09	6.38		9.65		.001	
**Time of viewing, n (%)**									.24
	Working time	236 (37.9)		33 (44.0)	203 (37.0)				
	Nonworking time	387 (62.1)		42 (56)	345 (63.0)				
	Weekday evening	188 (30.2)		22 (29.3)	166 (30.2)				
	Weekend/holiday	179 (28.7)		18 (24.0)	161 (29.4)				
	Weekday, weekend, and holiday overnight (12 AM to 5 AM)	20 (3.2)		2 (2.7)	18 (3.3)				

^a^N/A: not applicable.

**Table 4:** Contents of mini course videos viewed by junior and senior high students.

Category	Total (N=623), n (%)	Juniors (n=75), n (%)	Seniors (n=548), n (%)	*P* value
Emotional assistance	245 (39.3)	19 (25.3)	226 (41.2)	.008
Personal growth	229 (36.8)	18 (24.0)	211 (38.5)	.02
Family member relationship	42 (6.7)	18 (24.0)	24 (4.4)	<.001
Youth mental health	30 (4.8)	2 (2.7)	28 (5.1)	.35
Interpersonal relationship	10 (1.6)	4 (5.3)	6 (1.1)	.006
Others	67 (10.8)	14 (18.7)	53 (9.7)	.02

### Crisis Interventions Provided to Students at Risk of Suicide and Self-Injury

During the probation period, the current DMHC received three calls with suicide and self-injury risks. They were all from senior high school students. One of the calls was made during weekday lunch time and the other two were made on weekday evenings. The average duration of the three calls was 81 minutes. The DMHC staff provided timely crisis interventions for the three adolescents with suicidal and self-harm tendencies. Textbox 1 provides a synopsis of reported critical incidents and interventions provided by professionals linked to the DMHC for the three cases.

**Three case scenarios of critical incidents and corresponding interventions offered by the professional through the digital mental health clinic.**

- Case 1: This audio call came from a senior high school student during their lunch break (12:17-13:14 PM) lasting for 55.18 minutes.

*Caller:* I woke up in the morning with suicidal thoughts, and it has been strong until now. It was only after I am here in the phone booth that I began to feel better. When a headache hit me, I might look calm outside. In fact, I had lots of thoughts rushing through my mind. Part of me was trying to not commit suicide, another part of me said it would be all over if I die. It will be great if I can find relief for my emotions.

*Interventions*: After listening attentively, the psychologist encouraged the student to use relaxation techniques to relieve emotions. The psychologist also recommended that the student see a psychiatrist and follow the doctor’s advice on both medication and psychological counseling.

- Case 2: This audio call came from a senior high school student in the evening (7:40-9:22 PM) lasting for 102 minutes.

*Caller*: I have a history of depression, self-harming behaviors, and suicidal thoughts. Recently, I have been having a hard time controlling myself emotionally. I also have nightmares.

*Interventions*: After extending a series of emotional support techniques, including listening, affirmation, caring curiosity, and comfort, the psychologist helped the client sort through some of the cognitive issues. In the end, the psychologist advised the student to seek medical help.

- Case 3: This audio call came from a senior high school student in the evening (8:36-9:09 PM) lasting for 32 minutes.

*Caller*: I feel irritated, along with pressure on my chest and hard to breathe in crowded places. With certain things, I simply can’t force myself to be interested, including learning, and I am just not motivated. I tend to find pleasure in hurting myself, such as pinching my wounds. I have been contemplating on cutting myself with a knife but have not done so. I hope to improve my irritable mood when I am in a crowded place, but I don’t know how.

*Interventions:* After attentive listening, the psychologist utilized calming and affirmation techniques to provide comfort, in addition to asking if the student had been under tremendous pressure recently. She then advised the student to find the factors contributing to the student’s excessive stress. Following the advice, the psychologist indicated that the student could call back and discuss how to cope with the pressure and emotional challenges.

## Discussion

### Principal Findings

This study introduced and evaluated the multiple functions and effectiveness of a DMHC placed in secondary schools for students to access around the clock and in private. The DMHC was found to be effective in helping to connect the students with mental health needs to both human and digital resources during and after regular working hours. As a DHI tool, the current DMHC is well-aligned with the two themes of an established framework [13]: intervention-specific influences (acceptability, usability, and suitability) and person-specific influences (motivation, opportunity, and capability).

### Effectiveness

With its live video and audio call functions, the trial period witnessed a ratio of 5 calls/100 students with an average duration of 18 minutes and the majority of these calls were made during nonworking hours. In these calls, learning difficulties, interpersonal relationship problems, and emotional distress were the topics of greatest concern. The mini course video offered with the DMHC drew 9.1 views per 100 students, with the majority of the views occurring during nonworking hours, and emotional assistance and personal growth as the top topics of interest. This DMHC has a unique advantage in providing a space for timely access to crisis intervention. In addition, this DMHC proved to be an effective complementary resource for professional mental health service providers, especially during their down time.

### Live Call Usage

As seen in Table 1, junior and senior high students showed differences in making live calls. First, senior high school students made many more live calls and the calls lasted for a longer average duration as compared to junior high students. This difference can be attributed to two reasons. One is that senior high students faced greater academic pressure as they approached the national college entrance exam time and thus had higher level of needs for mental health assistance. This explains why more senior (32%) than junior (18%) high students sought help because of learning difficulties, as shown in Table 2. The other reason is that the majority of senior high students lived on campus throughout the week, including weekends, whereas only approximately 10% of the junior high students boarded at school. Second, a higher proportion of live calls from junior high students occurred during regular business hours (67.4%), whereas a higher percentage of calls from senior high students (61.3%) occurred during nonbusiness hours. Again, this difference may be attributed to greater academic pressure experienced by senior high students relative to the junior high students. Third, senior high students demonstrated a stronger preference for audio over video calls compared to junior high students (85.7% vs 72.3%). This confirms that adolescents prefer a channel where they could have their privacy and anonymity protected. The audio calling feature helps make them feel safe while talking to a professional from a trustworthy source.

### Reasons for Live Calls

Reasons for the live counseling calls fall mainly into three categories, as shown in Table 2: learning difficulties (31%), interpersonal relationships (27%), and emotional distress (29%). Consistent with prior literature [22], academic pressure/learning difficulty is the top challenge Chinese youth face, especially senior high students (32%). Therefore, interventions for senior high students should focus more on learning difficulties. In contrast to senior high students, half of the junior high students reported challenges with interpersonal (42%) and family (9%) relationships, which are closely related to emotional distress (30%). This calls for the attention of school mental health professionals to address the social emotional learning needs of junior high students. Importantly, some students complained about having to go to school on weekends and not being able to stay home and rest. To address this issue, a “double reduction” policy has been initiated and implemented in all elementary and secondary schools of the country, which aims to reduce the pressure placed on students due to excessive homework and after-school tutoring.

### Mini Course Video Usage

As seen in Table 3, a higher proportion of senior high students viewed the mini course videos compared to junior high students (9.65% vs 6.38%) and most of the views occurred during nonbusiness hours (63% vs 37% for working hours). The fact that more senior high students live on campus explains the greater overall use as well as the higher number of views during nonbusiness hours. Isolation from parents and/or family members increases the need for emotional assistance among senior high students, reflected by the fact that 41.2% of the mini course videos they viewed were about emotional assistance. In addition, senior high students are in the fastest stage of personal growth both physically and psychologically. Therefore, they have more concerns about their personal growth, as indicated by the higher proportion (38.5% vs 24%) of videos about personal growth viewed by seniors than juniors (Table 4).

Compared to senior high students, junior high students reported a more balanced need in three areas: emotional assistance (25%), personal growth (24%), and family relationships (24%). This again resonates with the findings from analysis of the reasons for the live calls, pointing to the importance of social emotional learning in this population. The overall higher level of mini course usage during nonbusiness hours (62%) in junior high students provides strong evidence for the value of the current DMHC in providing mental health prevention and intervention services to this population.

### Comparison With Other Available DHIs

Systemic searches of the literature were performed through the PubMed and Google Scholar databases, using the keywords “digital” OR “online” OR “internet based” AND “mental health” AND “services” OR “clinics” OR “interventions,” which retrieved studies of four similar mental health services [23]. Of these extant DHIs, Kindertelefoon is a free child helpline in Dutch and provides both online chat and telephone service to children aged between 8 and 18 years by means of a toll-free telephone number and an online chat app [24]. San Francisco–based Modern Health [25] offers several evidence-based modalities of care, including one-on-one care with mental health professionals for teletherapy, telecoaching, or both. This platform requires an appointment ahead of time for 30 to 50–minute sessions and is not free. Emohaa [23] and XiaoE [26] are two similar apps available in China offering live chat with artificial intelligence–based technologies.

However, since our DMHC is delivered on campus in dedicated and isolated booths, unlike most other DHIs, it offers numerous advantages outlined in Textbox 2, which supports the influencing factors defined in the framework of Liverpool et al [16].

There are also notable limitations of this DMHC, including the lack of tracking and personal history data gathering for the sake of maintaining anonymity. As a result, the tool does not facilitate building a relationship between the user and mental health care provider.

**Advantages of the digital mental health clinic (DMHC) corresponding to the influencing factors of digital health interventions (in parentheses) proposed in the framework of Liverpool et al [16].**

- Cost (accessibility & capability)

The current DMHC is provided at no cost to users and thus removes the financial barriers to seeking care.

- Privacy (acceptability & opportunity)

This DMHC does not collect visitors’ personal identification. Meanwhile, it keeps the visits anonymous, which are conducted in isolated and dedicated booths to ensure privacy.

- Access (usability & capability)

The DMHC is available around the clock. The students can be connected to mental health professionals and obtain audio/video information. This on-demand feature provides flexibility to students who are constrained by availability of a phone or computer, time conflict with school schedules, parents’ availability to take them to physical clinics, or simply lack of mental health care professionals, among other barriers to access. This is especially important in a crisis such as suicidal attempts or in remote areas with minimal mental health care resources.

- Focuses on students (suitability & motivation)

This DMHC was designed with adolescents in mind. The counselors are trained in children’s psychology and the contents in the audio and video resources reflect the most important issues facing youth. It is also user-friendly and students can navigate the interface without difficulty.

- Self-directed help (acceptability & opportunity)

The students who use this tool can choose the level of therapist involvement, ranging from no involvement to one-on-one consultation. Fischer and Turner [27] identified resistance of seeking professional services to address a personal crisis as one critical factor deterring youth from seeking formal help. Instead, they turn to friends and family, or rely on themselves to obtain informal help [21]. As compared to the four platforms under review, our DMHC provides the most functionality. The Kindertelefoon, Emohaa [23], and XiaoE [26] digital health interventions offer chat-only services. Modern Health provides teletherapy, telecoaching, as well as education, but the education provided by Modern Health is one-on-one rather than through asynchronous materials.

### Future Directions for Development of the DMHC

In preparation for a scalable next step, features of the current DHI can be enhanced with the following considerations. First, providing options for students who are willing to leave contact information or personal identification marks for a long-term service. Second, close follow-ups should be recommended for students with suicidal and self-injury risks. In the long-run, the DMHC can be integrated with a stepped-care model to facilitate identification, tracking, referral, and treatment of students with different levels of mental health risks. Third, based on the challenges students reported during the audio and video calls, the design team may consider adding to the contents of mini courses related to students’ learning difficulties and interpersonal relationships, while strengthening its current resources on personal growth and emotional stress. Fourth, future design may also consider adding structured surveys, including a satisfaction survey; instruments on school refusal; measures on depression, stress, and anxiety; as well as demographic information. This will shed light on the characteristics and needs of the adolescents using the services and lead to enhanced service features and quality. Fifth, adopting new technological platforms such as a mobile app will improve the accessibility and potential interactiveness of this mental health tool. Additionally, incorporating natural language processing will further enhance the experience in the interaction process.

### Conclusion

This DMHC has demonstrated capability in addressing the mental health needs of adolescents, especially during nonbusiness hours as a supplementary solution. As a prevention tool, it provides students opportunities to connect with a mental health professional and educational materials to help them assess their mental states and enhance their knowledge of mental health. In times of crisis, it can also be an effective intervention tool through its ability to quickly respond to mental health emergencies.

## Abbreviations

DHI: digital health intervention
DMHC: digital mental health clinic
e-Hospital: electronic hospital

## Data Availability

The data that support the findings of this study are available on request from the corresponding author.

## References

[1] Perou R, Bitsko RH, Blumberg SJ, Pastor P, Ghandour RM, Gfroerer JC, Hedden SL, Crosby AE, Visser SN, Schieve LA, Parks SE, Hall JE, Brody D, Simile CM, Thompson WW, Baio J, Avenevoli S, Kogan MD, Huang LN, Centers for Disease Control and Prevention (CDC) (2013). Mental health surveillance among children--United States, 2005-2011. MMWR Suppl.

[2] Polanczyk GV, Salum GA, Sugaya LS, Caye A, Rohde LA (2015). Annual research review: a meta-analysis of the worldwide prevalence of mental disorders in children and adolescents. J Child Psychol Psychiatry.

[3] Zheng Y (2015). Development and prospects of child psychiatry in China. Chinese J Psychiatry.

[4] Chi X, Liang K, Chen S, Huang Q, Huang L, Yu Q, Jiao C, Guo T, Stubbs B, Hossain MM, Yeung A, Kong Z, Zou L (2021). Mental health problems among Chinese adolescents during the COVID-19: the importance of nutrition and physical activity. Int J Clin Health Psychol.

[5] Costello EJ, He J, Sampson NA, Kessler RC, Merikangas KR (2014). Services for adolescents with psychiatric disorders: 12-month data from the National Comorbidity Survey-Adolescent. Psychiatr Serv.

[6] Shapiro M (2018). Teenage depression: if a parent doesn't get treatment for a child, is that abuse?. The Conversation.

[7] Copeland WE, Angold A, Shanahan L, Costello EJ (2014). Longitudinal patterns of anxiety from childhood to adulthood: the Great Smoky Mountains Study. J Am Acad Child Adolesc Psychiatry.

[8] Wu J, Pan J (2019). The scarcity of child psychiatrists in China. Lancet Psychiatry.

[9] Shi Q (2018). School-based counseling in mainland China: past, present, and future. J Sch Based Counsel Policy Eval.

[10] Gulliver A, Griffiths KM, Christensen H (2010). Perceived barriers and facilitators to mental health help-seeking in young people: a systematic review. BMC Psychiatry.

[11] Samargia LA, Saewyc EM, Elliott BA (2006). Foregone mental health care and self-reported access barriers among adolescents. J Sch Nurs.

[12] Andrews G, Cuijpers P, Craske MG, McEvoy P, Titov N (2010). Computer therapy for the anxiety and depressive disorders is effective, acceptable and practical health care: a meta-analysis. PLoS One.

[13] Anthes E (2016). Mental health: there's an app for that. Nature.

[14] Chandrashekar P (2018). Do mental health mobile apps work: evidence and recommendations for designing high-efficacy mental health mobile apps. Mhealth.

[15] Khanna MS, Carper M (2022). Digital mental health interventions for child and adolescent anxiety. Cogn Behav Pract.

[16] Liverpool S, Mota CP, Sales CMD, Čuš A, Carletto S, Hancheva C, Sousa S, Cerón SC, Moreno-Peral P, Pietrabissa G, Moltrecht B, Ulberg R, Ferreira N, Edbrooke-Childs J (2020). Engaging children and young people in digital mental health interventions: systematic review of modes of delivery, facilitators, and barriers. J Med Internet Res.

[17] Clark LH, Hudson JL, Dunstan DA, Clark GI (2020). Barriers and facilitating factors to help‐seeking for symptoms of clinical anxiety in adolescent males. Austral J Psychol.

[18] Ba H, Tally W, Tsikalas K (2002). Investigating children's emerging digital literacies. J Technol Learn Assess.

[19] Blummer B (2017). Digital literacy practices among youth populations: a review of the literature. Educ Lib.

[20] Podina IR, Mogoase C, David D, Szentagotai A, Dobrean A (2015). A meta-analysis on the efficacy of technology mediated CBT for anxious children and adolescents. J Rat Emot Cogn Behav Ther.

[21] Wang C, Barlis J, Do KA, Chen J, Alami S (2019). Barriers to mental health help seeking at school for Asian– and Latinx–American adolescents. School Mental Health.

[22] Wang C, Zhang P, Zhang N (2020). Adolescent mental health in China requires more attention. Lancet Public Health.

[23] Sabour S, Zhang W, Xiao X, Zhang Y, Zheng Y, Wen J, Zhao J, Huang M (2023). A chatbot for mental health support: exploring the impact of Emohaa on reducing mental distress in China. Front Digit Health.

[24] Fukkink RG, Hermanns JMA (2009). Children's experiences with chat support and telephone support. J Child Psychol Psychiatry.

[25] Prescott MR, Sagui-Henson SJ, Welcome Chamberlain CE, Castro Sweet C, Altman M (2022). Real world effectiveness of digital mental health services during the COVID-19 pandemic. PLoS One.

[26] He Y, Yang L, Zhu X, Wu B, Zhang S, Qian C, Tian T (2022). Mental health chatbot for young adults with depressive symptoms during the COVID-19 pandemic: single-blind, three-arm randomized controlled trial. J Med Internet Res.

[27] Fischer EH, Turner JI (1970). Orientations to seeking professional help: development and research utility of an attitude scale. J Consult Clin Psychol.

